# A Mutant Methionyl-tRNA Synthetase-Based toolkit to assess induced-Mesenchymal Stromal Cell secretome in mixed-culture disease models

**DOI:** 10.21203/rs.3.rs-2838195/v1

**Published:** 2023-05-03

**Authors:** Jeremy D. Burgess, Danilyn Amerna, Emily S. Norton, Tammee M. Parsons, Ralph B. Perkerson, Ayman H. Faroqi, Zbigniew K. Wszolek, Hugo Guerrero Cazares, Takahisa Kanekiyo, Marion Delenclos, Pamela J. McLean

**Affiliations:** Mayo Clinic Graduate School of Biomedical Sciences; Mayo Clinic Jacksonville Campus: Mayo Clinic in Florida; Mayo Clinic Graduate School of Biomedical Sciences; Mayo Clinic Jacksonville Campus: Mayo Clinic in Florida; Mayo Clinic Jacksonville Campus: Mayo Clinic in Florida; Mayo Clinic Graduate School of Biomedical Sciences; Mayo Clinic in Florida Department of Neurology and Neurosurgery; Mayo Clinic in Florida Department of Neurology and Neurosurgery; Mayo Clinic Jacksonville Campus: Mayo Clinic in Florida; Mayo Clinic Jacksonville Campus: Mayo Clinic in Florida; Mayo Clinic Jacksonville Campus: Mayo Clinic in Florida

**Keywords:** Mesenchymal stromal cells (MSCs), Secretome, Cell-type specific proteomics, Mixed-culture disease models, Mutant methionyl-tRNA synthetase (MetRSL274G), Bioorthogonal non-canonical amino acid tagging (BONCAT), Azidonorleucine (ANL), CRISPR/Cas9, Induced-MSCs (iMSCs)

## Abstract

**Background:**

Mesenchymal stromal cells (MSCs) have a dynamic secretome that plays a critical role in tissue repair and regeneration. However, studying the MSC secretome in mixed-culture disease models remains challenging. This study aimed to develop a mutant methionyl-tRNA synthetase-based toolkit (MetRS^L274G^) to selectively profile secreted proteins from MSCs in mixed-culture systems and demonstrate its potential for investigating MSC responses to pathological stimulation.

**Methods:**

We used CRISPR/Cas9 homology-directed repair to stably integrate MetRS^L274G^ into cells, enabling the incorporation of the non-canonical amino acid, azidonorleucine (ANL), and facilitating selective protein isolation using click chemistry. MetRS^L274G^ was integrated into both in H4 cells and induced pluripotent stem cells (iPSCs) for a series of proof-of-concept studies. Following iPSC differentiation into induced-MSCs, we validated their identity and co-cultured MetRS^L274G^-expressing iMSCs with naïve or lipopolysaccharide- (LPS) treated THP-1 cells. We then profiled the iMSC secretome using antibody arrays.

**Results:**

Our results showed successful integration of MetRS^L274G^ into targeted cells, allowing specific isolation of proteins from mixed-culture environments. We also demonstrated that the secretome of MetRS^L274G^-expressing iMSCs can be differentiated from that of THP-1 cells in co-culture, and is altered when co-cultured with LPS-treated THP-1 cells compared to naïve THP-1 cells.

**Conclusions:**

The MetRS^L274G^-based toolkit we have generated enables selective profiling of the MSC secretome in mixed-culture disease models. This approach has broad applications for examining not only MSC responses to models of pathological conditions, but any other cell type that can be differentiated from iPSCs. This can potentially reveal novel MSC-mediated repair mechanisms and advancing our understanding of tissue regeneration processes.

## I. Background

Mesenchymal stromal cells (MSCs) are multipotent adult stem-like cells thought to exert their beneficial effects through paracrine secretion of factors, including proteins, rather than direct engraftment at the site of damage(1–3). While studies suggest the dwell time of MSCs at the site of damage is relatively brief(4), the secretome of MSCs is dynamic and can be influenced by the microenvironment, including the presence of pathology(5–7).

Significant insight into pathogenic mechanisms and potential therapeutic targets can be garnered by examining the response of MSCs when co-cultured with cellular models of disease. A major challenge associated with this approach is the limited resolution of proteomic methodologies to detect low abundance secreted proteins(8). In the co-culture scenario, this is exacerbated by reduced signal to noise due to competing secretions from both cell types as well as contributions from media components. Non-canonical amino acid tagging (NCAT) approaches have traditionally been used to enrich proteomes on a temporal basis(9, 10) but advances in the approach now permit enrichment on a cell-specific basis(11, 12). Engineered mutant methionyl-tRNA synthetase (MetRS^L274G^) can utilize azidonorleucine (ANL) as an alternative substrate in place of methionine during protein translation(13). When ANL is incorporated into proteins, the conferred azide moiety can be targeted for labeling or isolation using click chemistry approaches. Since wildtype MetRS cannot incorporate ANL, only proteins translated in those cells expressing the mutant form are tagged.

Recent studies have utilized MetRS^L274G^-mediated protein tagging to characterize human placenta- and murine bone marrow-derived MSC secretomes(14, 15) from mixed cell environments, however they rely on lentiviral transduction to mediate MetRS^L274G^ expression in the MSCs. Such an approach requires extremely high transduction efficiency and/or uses valuable, potent, early passages of MSCs to select for MetRS^L274G^ expressing cells. Furthermore, because lentiviral transduction does not allow for targeted transgene insertion there is a risk of genomic disruptions due to transgene integrations.

Here we have devised a targeted gene editing approach to generate cells stably expressing MetRS^L274G^ at the Citramalyl-CoA Lyase (*CLYBL*) safe harbor locus(16) to avoid issues from off-target insertions. Further, we have used this strategy to generate MetRS^L274G^-expressing iPSCs and subsequently differentiated them to induced-MSCs (iMSCs) to overcome potential issues with passage number, potency, and donor variability that come with the use of primary MSCs(17, 18). Herein we validate our gene editing approach first in H4 neuroglioma cells, and subsequently in induced pluripotent stem cell (iPSC)-derived induced MSCs. Using H4 neuroglioma cells stably expressing MetRS^L274G^ we demonstrate the specific isolation of secreted products from co-culture with non-MetRS^L274G^-expressing cells. We further apply this innovative technology to iPSCs, differentiating them to iMSCs and demonstrating the selective isolation of secreted proteins from MetRS^L274G^-expressing iPSC-derived-iMSCs from media of co-cultured cells allowing comparison of iMSC secretomes in response to different co-cultured conditioning stimuli. We demonstrate the utility of these MetRS^L274G^-expressing iMSCs to explore changes induced in iMSC secretome when cultured with either naïve, or lipopolysaccharide- (LPS) treated THP-1 cells, as a model of an inflammatory environment.

## II. Methods

### Generation of MetRS^L274G^ donor plasmid:

For subcloning, all restriction enzymes were purchased from New England Biolabs. Bands were detected in 1% agarose (Genessee, #20–102GP) gels using SmartGlow Pre Stain (Accuris, #E4500-PS), excised, and purified with Zymogen Gel DNA recovery kit (#11–300C). Ligations used T4 ligase (NEB), and transformations were carried out with NEB^®^ 5-alpha Competent E. coli (High Efficiency) according to manufacturer’s protocol.

CLYBL-TO-hNGN2-BSD-mApple, a gift from Michael Ward (Addgene plasmid # 124229; http://n2t.net/addgene:124229; RRID:Addgene_124229), was digested at the NotI and PacI sites to remove all but the CLYBL homology arms and bacterial origin of replication, re-ligated, incorporating a custom designed multiple cloning site (MCS). The custom MCS was annealed from oligos /5Phos/GGCCACGCGTGCGGCCGCACTAGTATTTAAATCACTACGTGGCTAGCGTCGACTTATAATTAAT and /5Phos/TAATTATAAGTCGACGCTAGCCACGTAGTGATTTAAATACTAGTGCGGCCGCACGCGT (IDT). This new plasmid will be referred to as CLYBL-MCS.

CLYBL-MCS was then used in two parallel reactions. NotI and SpeI digestion was used to move the BSD-mApple selection cassette from the original CLYBL-TO-hNGN2-BSD-mApple plasmid into CLYBL-MCS, to make CLYBL-MCS-BSD-mApple. Concurrently, PsiI and SalI digestion was used to move the CAG promoter from the original CLYBL-TO-hNGN2-BSD-mApple plasmid into a second copy of CLYBL-MCS. Downstream of this, NheI and DraIII digestion of pMarsL274G - a gift from David Tirrell (Addgene plasmid # 63177; http://n2t.net/addgene:63177; RRID:Addgene_63177) was used to insert the L274 mutation-containing mouse *Mars* gene. Finally, PacI and SwaI restriction sites were used to transfer the new CAG-MarsL274G region into the CLYBL-MCS-BSD-mApple plasmid, creating CLYBL-BSD-mApple-CAG-MetRS^L274G^.

A version of this plasmid was also generated in which the LoxP-flanked BSD-mApple selection casette was replaced with a Puromycin-eGFP(NLS) version. A new MCS, incorporating LoxP sites was generated in two steps. First, /5Phos/GGCCACGCGTataacttcgtataatgtatgctatacgaagttatGGCGCGCCTTAAT and /5Phos/TAAGGCGCGCCataacttcgtatagcatacattatacgaagttatACGCGT (IDT) were annealed and inserted into CLYBL-TO-hNGN2-BSD-mApple between NotI and PacI sites (CLYBL_Adapt2). In parallel, /5Phos/GGCCGGCGCGCCGCTCTTCGGCTataacttcgtataatgtatgctatacgaagttatATTTAAATTTAAT and /5Phos/TAAATTTAAATataacttcgtatagcatacattatacgaagttatAGCCGAAGAGCGGCGCGCC were annealed and inserted into CLYBL-TO-hNGN2-BSD-mApple, also between NotI and PacI sites (CLYBL_Adapt3). The region between PacI and AscI of CLYBL_Adapt3 was inserted into CLYBL_Adapt2 to make CLYBL_Adapt2+3. A custom plasmid containing T2A-linked Puromycin resistance element and eGFP with nuclear localization signal, driven by EF1α promoter was ordered from VectorBuilder (VB210430–1160qqx), and the EF1α-Puro-T2A-(NLS)eGFP portion excised with AscI and SapI and inserted into CLYBL_Adapt2+3 (CLYBL_Adapt2+3_EF1α-Puro-T2A-(NLS)eGFP). Finally the selection cassette in CLYBL-BSD-mApple-CAG-MetRS^L274G^ was replaced with the one from CLYBL_Adapt2+3_EF1α-Puro-T2A-(NLS)eGFP, using SwaI and MluI to create CLYBL-Puro-(NLS)eGFP-CAG-MetRS^L274G^.

### Generation of stable H4.MetRS^L274G^ cells with CRISPR/Cas9 guided homology directed repair (HDR):

H4 cells throughout this manuscript are derived from H4 HTB-148 cells (ATCC). Unmodified H4 cells are referred to henceforth as H4.WT. All H4 cells were cultured in OptiMEM, reduced serum medium supplemented with GlutaMAX (Gibco, #51985–034), with 10% fetal bovine serum (FBS) (Gibco, #10437–028) unless otherwise stated. Cells were cultured at 37°C in 5% CO_2_, and passaged using 0.25% trypsin/EDTA (Gibco, #25200–056) upon reaching confluence (every ~5 days).

To generate H4 cells stably expressing MetRS^L274G^, cells were seeded in 6-well plates at 2.5e5 cells per well in standard culture media. After 24 hours cells were washed with DPBS and supplemented with fresh media. Transfection mixes were prepared. Custom CRISPRevolution sgRNA EZ Kit (Synthego) to target the CLYBL safeharbor site (AUGUUGGAAGGAUGAGGAAA) was ordered and 1μM sgRNA was complexed with 1μM Alt-R HiFi Cas9 Nuclease (IDT) in 125μL OptiMEM (no serum) per well for 5 minutes at room temperature. 500ng donor plasmid, CLYBL-Puro-(NLS)eGFP-CAG-MetRS^L274G^ or CLYBL-BSD-mApple-CAG-MetRS^L274G^, and 100ng dominant negative p53 plasmid (pCE-mp53DD, a gift from Shinya Yamanaka (Addgene plasmid # 41856; http://n2t.net/addgene:41856; RRID:Addgene_41856) to increase survival in cells undergoing double-strand breaks, were added to the RNP complex and mixed with 10μL Lipofectamine 2000 (Invitrogen, #11668–019) in 125μL OptiMEM per well for a total transfection mix volume of 250μL per well. This was added dropwise to the prepared wells and incubated overnight.

After 24 hours, media was exchanged for fresh, and after a further 24 hours, antibiotic selection was initiated with 10μg/mL blasticidin (Invivogen, #ant-bl-05) or 5μg/mL puromycin (Invivogen, #ant-pr-1). When cells were approaching confluence, they were diluted to 10 cells/mL and plated in 100μL media in a 96-well plate to generate clones from single cells. After 7 days, wells with single colonies displaying the expected fluorescence were passaged to 24-well plates for expansion, then screened by FUNCAT labeling, PCR, and qPCR as described below. Clones passing screening (PCR and FUNCAT described subsequently) were passaged to T25 flasks for expansion, cryopreservation, and use in future experiments. To excise the selection cassette, H4.MetRS^L274G^ cells were plated in standard media conditions at 50,000 cells per well in 24-well plates. After 24 hours they were washed with DPBS and treated with 5μM TAT-CRE (Millipore-Sigma) in standard media for 6 hours. Cells were washed twice with DPBS and cultured for 2–3 days in standard media. Upon reaching confluence, cells were passaged using 0.25% trypsin/EDTA and serially diluted across a 6-well plate. Non-fluorescing clonal colonies were manually picked under an EVOS fluorescent microscope and transferred to a 48-well plate. Once colonies were established, they were expanded to 6 well-plates. Clones were screened for continued lack of fluorescence and efficient FUNCAT labeling, then PCR tested to detect predicted band following deletion of selection cassette. Clones that passed FUNCAT and PCR validation were expanded and frozen for use in further experiments.

### Polymerase chain reaction (PCR) to confirm on-target transgene insertion:

Genomic DNA was extracted from cells according to manufacturer’s protocols using either Wizard^®^Genomic DNA Purification Kit (Promega, #A1120) or QuickExtract (Lucigen, #QE09050). PCR reactions were carried out using 2X GoTaq (Promega, #M7123) with custom primer pairs (IDT). To confirm MetRS^L274G^ transgene integration: fwd-ACCGGAGCCCAACTTTTCT/rev- TTGTTCTTCCCCAAGTCTTTCT. To confirm selection cassette excision: fwd-TTAGTGCTTTACGGCACCTC/rev- TTGTTCTTCCCCAAGTCTTTCT.

### Fluorescent Non-Canonical Amino Acid Tagging (FUNCAT) with Cy5-DBCO:

MetRS^L274G^ or WT cells were treated with 4mM ANL (Iris Biotech, #HAA1625.0001) or vehicle in complete media for 18 hours. Media was aspirated and the cells twice rinsed with ice-cold PBS, pH 7.4 containing 1mM MgCl_2_ and 0.1mM CaCl_2_. Cells were fixed in 4% PFA for 20 minutes at room temperature, prior to 3× 10-minute washes with PBS, pH 7.4. Cells were blocked and permeabilized for 1 hour at room temperature in 10% normal horse serum, 5% sucrose, 2% bovine serum albumin (BSA), 0.1% triton-X in PBS, pH 7.4, then washed 3× 10 minutes in PBS, pH 7.8. Cells were stained 1 hour at room temperature in the dark with 500nm Cy5-DBCO (Click Chemistry Tools, #A130–1) in PBS, pH 7.8. Cells were washed 2x for 10 minutes in 1% tween-20, 0.5mM EDTA in PBS, pH7.8, followed by 3× 5minutes in PBS, pH 7.4. Nuclei were counterstained using 1:10,000 Hoechst 33342 (Invitrogen, #H3570) during the second of these final 3 washes. Stained cells were imaged using an Axio Observer Z1 inverted microscope (Carl Zeiss, Germany) or LSM800 confocal microscope (Zeiss).

### BONCAT pipeline with H4 cell line lysate:

H4 neuroglioma cells expressing mCherry (henceforth referred to as H4.mCherry), or H4.MetRS^L274G^.eGFP cells were cultured both separately and in co-culture in OptiMEM + 10% FBS. Cultures were treated for 72 hours with 4mM ANL or vehicle. Cells were rinsed and scraped in PBS before lysis with triton-x-based lysis buffer (150mM NaCl, 50mM Tris, 0.1% Triton X-100) with protease inhibitor (Roche, #4693132001). All steps were carried out in low protein binding tubes (Pierce). Total protein was calculated using BCA assay (Pierce) and 1500μg protein from each culture and treatment group was suspended in PBS, pH 7.8, alkylated with 50mM iodoacetamide (IA; Sigma, #I1149–5G) for 30 minutes in the dark at room temperature while mixing at 1100rpm, and biotinylated using 10μM DBCO-S-S-Peg3-Biotin (BroadPharm, #BP-22453) for 1 hour at room temperature while mixing at 1100rpm. Excess DBCO-S-S-Peg3-Biotin was removed using PD Spintrap G-25 desalting columns (Cytiva Life Sciences, #28918004) and biotinylated proteins were bound to 100μL Streptavidin Sepharose High Performance Beads (Cytiva Life Sciences, #17511301) overnight at 4°C in binding buffer (20 mM sodium phosphate, 0.15 M NaCl, pH 7.5, 0.05% SDS) under rotation. The following day, beads were transferred to 2mL Pierce Disposable Columns and washed 3 times with 2mL dH_2_O, 10 times with 2mL 1% SDS in PBS, pH 7.4, and 5 times in PBS, pH 7.4. Beads were then transferred to Costar Spin-X Centrifuge Tube Filters (Corning Life Sciences, #8161) in 500μL PBS and spun twice for 1 minute at 1000G to remove the liquid. Flow-through was aspirated and the beads resuspended in 100μL elution buffer (50mM dithiothreitol (DTT) and 1X protease inhibitor in PBS) and shaken for 4 hours at 37°C. Tube filters were twice spun for 1 minute at 1000G and the flow through (eluate) collected and aliquoted for freezing at −80°C until western blotting and silver stains.

### H4.mCherry and H4.MetRS^L274G^.eGFP cell images

Images of these cells were captured using an EVOS-FL fluorescent microscope (Invitrogen) with 10X objective and GFP and RFP light cubes.

### Silver stains

30μL BONCAT eluate was mixed with 11.5μL 4X NuPAGE LDS Sample Buffer (Thermofisher Scientific, #NP0007) and 4.6μL 10X NuPAGE Sample Reducing Agent (Thermofisher Scientific, #NP0009). Samples were heated to 70°C for 10 minutes then returned to room temperature and loaded into Bolt 4–12% Bis-Tris Gels (Thermofisher Scientific, #NW04120BOX) and run in ice-cold 1X BOLT MOPS SDS running buffer (Thermofisher Scientific, #B0001) at constant 200V for ~50 minutes. Gels were silver stained according to the manufacturer’s protocol using Pierce Silver Stain Kit (Thermofisher Scientific, #24612). In brief, gels were washed in ultrapure water, fixed with 30% ethanol, 10% acetic acid in dH_2_O, washed in 10% ethanol, then ultrapure water. Gels were sensitized with sensitizer solution, before being briefly washed with dH_2_O and stained for 30 minutes with stain working solution. Gels were briefly washed with dH_2_O and developed with developer working solution and once optimal signal was observed, stopped with 5% acetic acid in dH_2_O. Stained gels were imaged using the Silver Stain Setting on a Chemidoc MP (Bio-Rad).

### Western blots

30μL eluate, 30μg pre-BONCAT protein lysate, or 10μL pre-BONCAT concentrated CM was prepared with a fluorescence-compatible 4X laemelli loading buffer (125mM Tris-HCl, pH 6.8, 50% glycerol, 4% SDS, 0.2% Orange G, with 1:10 beta-mercaptoethanol (Bio-Rad, #1610710)) and heated at 95°C for 5 minutes before loading into 10-well Any kD Mini-PROTEAN TGX Stain-Free gels (Bio-Rad, # 4568124). Ladder was 2μL Precision Pus Protein Dual Color Standard (Bio-Rad, #1610374). Gels were run in 1X Tris/Glycine/SDS running buffer (Bio-Rad, #1610772) at 300V for ~21 minutes. Proteins were transferred to methanol-activated, 0.45μm low fluorescence (LF) PVDF membranes (Bio-Rad, #10026934) using Trans-Blot Turbo RTA Mini Transfer Kit (Bio-Rad) with Trans-Blot Turbo Transfer System (Bio-Rad) using the 7-minute mixed-molecular weight setting. Following transfer, proteins were further fixed to the membranes by drying before re-activating with methanol, twice rinsing with dH_2_O, and blocking for 1 hour at room temperature in 5% non-fat milk (LabScientific, #M0841) in TBS. Blocked membranes were incubated in primary antibodies as described in table 1 in 5% milk with 0.05% tween-20 in TBS overnight at 4°C. The following day membranes were washed 3 times for 5 minutes each in TBS-T (TBS with 0.05% tween-20) and incubated with secondary antibodies as described in table 1 for 1 hour at room temperature in 5% milk with 0.05% tween-20 in TBS. Blots were washed 3 times for 5 minutes each with TBS-T, followed by one 5-minute wash in TBS. For actin-HRP in the H4.mCherry and H4.MetRS^L274G^.eGFP lysate experiments, and FlexAble CoraLite^®^ 488-αsyn in fluorescent-αsyn transfected H4 CM experiments, those antibodies were added overnight at 4°C in 5% milk, 0.05% tween-20 in TBS after all other steps including washing following secondary antibody incubation for the other proteins in the blot had been completed. Membranes were then washed as described following secondary antibody incubation.

FlexAble CoraLite^®^ 488 labeling of anti-Alpha-synuclein antibody [MJFR1] was carried out using FlexAble CoraLite^®^ 488 Antibody Labeling Kit for Rabbit IgG kit (Proteintech) according to manufacturer’s instructions.

All blots were imaged using a Chemidoc MP on either IRdye 680, IRdye 800, or Alexafluor 488 settings depending on the secondary antibodies used for that particular blot.

### BONCAT pipeline with H4 cell conditioned media

H4.WT and H4.MetRS^L274G^ (without fluorescent selection cassette) were each plated separately in 15cm dishes for 24 hours before being transfected with expression plasmids for αsyn-RFP or αsyn-eGFP, respectively, using Effectene (Qiagen, #301425) transfection reagent according to manufacturer’s protocol. The following day cells were rinsed with DPBS and media replaced with OptiMEM (Gibco) supplemented with 4mM ANL. After 72 hours, media was collected, centrifuged for 10 minutes at 2500G to remove cells and debris, supplemented with protease inhibitor to 1X, and concentrated using Amicon-Ultra Centrifugal filter units with 10kDa molecular weight cutoff (MWCO) (Millipore Sigma, #UFC901024). Concentrated CM had further addition of protease inhibitor to 1X based on the new volume, as well as RIPA buffer (Millipore Sigma, #20–188) to 1X. CM was then sonicated for 5 cycles of 30 seconds on, 30 seconds off on the ‘high’ setting of a Bioruptor 300 (Diogenode) at 4°C. A small volume of each CM was set aside as pre-BONCAT samples. Of the remaining volumes, one third from each CM was combined to leave three samples of equal volume: H4.WT transfected with αsyn-RFP, H4.MetRS^L274G^ transfected with αsyn-eGFP, and a 50:50 mixture of the aforementioned 2 samples to simulate co-culture. These three CM samples then underwent the same BONCAT, affinity purification, and elution protocol as described previously for the H4 cell lysate experiments. Silver stains and WBs were performed on the pre-BONCAT samples and eluates according to the same protocols described for earlier experiments.

### Comparison of serum-containing vs serum-free media following BONCAT and affinity purification pipeline

H4.WT or H4.MetRS^L274G^ cells were plated separately in 15cm dishes and allowed to equilibrate for 24 hours. Media was then replaced with phenol red-free OptiMEM containing 4mM ANL and with or without 5% FBS supplementation. After 72 hours, media was harvested, concentrated, and subjected to the BONCAT and affinity purification pipeline described above. Silver stains were performed as described above to demonstrate the elimination of contaminating media components in post-BONCAT eluates.

### Generation of stable iPSC.MetRS^L274G^ cells with CRISPR/Cas9 guided homology directed repair (HDR):

iPSCs were generated from dermal fibroblasts from a control individual with informed consent, under the appropriate IRB protocols (details in Declarations section), and converted to iPSCs by electroporation with three plasmids to express OCT3/4, SOX2, KLF4, L-MYC, LIN28, and p53-shRNA. Seven days after nucleofection, cells were replated and cultured in mTeSR1 complete media (Stemcell Technologies, #85850) and colonies isolated and expanded for 3–4 weeks with daily media changes as previously described (37).

Following establishment of the iPSC lines, they were maintained in mTeSR Plus media (StemCell technologies, #100–0276) on Matrigel or Cultrex coated plasticware. Routine passaging of colonies was carried out using ReLeSR (StemCell Technologies, #05872), or Accutase (Innovative Cell Technologies, #NC9464543) where plating as single cells was preferred. Following passaging, media was supplemented for 24 hours with 10μM rock inhibitor Y-27632 (RI; StemCell Technologies, #72304). For cryopreservation, dissociated colonies were frozen using CryoStor CS10 (StemCell Technologies, #07930) or NutriFreez^®^ D10 without Phenol Red (Biological Industries, #05-714-1B).

MetRS^L274G^-expressing iPSCs were generated in a similar manner to that described for the H4 cells. Briefly, 80% confluent iPSCs were dissociated to a single cell suspension using Accutase and 2.5e5 cells plated in a well of a matrigel-coated 24-well plate in mTesR1 supplemented with 10μM RI. After allowing cells to settle for several hours, media was aspirated, cells rinsed with pre-warmed DMEM/F12 (Gibco, #11330032), and fresh mTeSR1 + 10μM RI added to eliminate residual Accutase and ensure optimal transfection conditions. After a further hour, HiFi Cas9, CLYBL sgRNA, CLYBL-Puro-(NLS)eGFP-CAG-MetRS^L274G^ donor plasmid, and pCE-mp53DD plasmid transfection complexes were prepared as described for the H4 cell procedure and added dropwise to the iPSCs. The following day, cells were rinsed with DMEM/F12 and fresh mTesR1 without RI added and supplemented with 0.2μg/mL puromycin. Cells were monitored over subsequent days and received fresh media containing puromycin each day. Next steps were dependent on cell line. Those that responded well to antibiotic selection and had a high enrichment of cells expressing green fluorescence when observed under the EVOS microscope were passaged as single cells using Accutase and serially diluted across a matrigel-coated 6-well-plate in RI-containing mTeSR1. Alternatively, for those cell lines that were less responsive to antibiotic selection, colonies or areas of colonies with abundant GFP expression when observed with EVOS microscope were manually picked from the growth surface using a pipette tip and consolidated into a matrigel-coated well of a 24-well plate. After allowing the picked colonies to recover, they were passaged as single cells using Accutase and serially diluted across a six well plate. Once single cells established themselves as clonal populations, fluorescing colonies were picked with a pipette tip and transferred to separate wells of 96-well plates. Once established, plates were passaged to two parallel 96-well plates using Accutase. One plate was incubated overnight with 4mM ANL before fixing and FUNCAT labeling with cy5-DBCO as previously described to identify colonies with functional MetRS^L274G^. Colonies displaying homogenous cy5 labeling during imaging using Operetta CLS were then passaged from their equivalent well in the parallel plate to two sister 24-well plates. One plate was used to extract DNA (QuickExtract; Lucigen) for PCR validation of on-target transgene insertion as described earlier. Functional colonies with correct transgene insertion were then expanded from the equivalent well in the sister 24-well plate and cryopreserved. In order to remove the selection casette selected, validated colonies were treated with recombinant TAT-CRE, before repeating the steps described to select clonal cell colonies that no longer had green fluorescence, still labeled with cy5 DBCO following ANL treatment, and had on target transgene insertion according to PCR analysis.

### Stably edited iPSC Quality Control

To ensure quality of our iPSCs, those clones with integrated MetRS^L274G^ and excised selection casettes underwent a battery of quality control procedures prior to use in any further experiments. Firstly, cells were sent to the Mayo Clinic Cytogenetics Core (Rochester, MN) for GTL-Banding karyotyping to confirm no chromosomal abnormalities had been introduced.

Pluripotency was confirmed by differentiation of selected clones to the three germ layers using StemXVivo Mesoderm, Endoderm, and Ectoderm kits (R&D systems) according to the manufacturers protocols. In brief iPSCs were plated in 24-well plates and treated with relevant differentiation medias from the kits at the timepoints specified in the protocol. Cells were fixed for immunostaining with 4% PFA in PBS at day 3 (mesoderm) or day 4 (endoderm and ectoderm). Cells were washed 3× 5 minutes with 1% BSA in PBS, then blocked and permeabilized for 45 minutes at room temperature with 0.3% triton X 100, 1% BSA, and 10% normal donkey serum in PBS. Cells were incubated overnight at 4°C in primary antibodies to validate endoderm, mesoderm, or ectoderm identity (anti-human SOX17, Brachyury, or Otx2, respectively). Cells were washed 3× 5 minutes in 1% BSA in PBS before incubation with secondary antibody (Alexafluor 488 donkey anti-goat IgG; Thermo Fisher Scientific, #A11055) for 1 hour at room temperature. Cells were washed 3× 5 minutes in 1% BSA in PBS, with 1:10000 Hoechst 33342 included in the penultimate wash. Immunolabelled cells were imaged using Axio Observer Z1 inverted microscope (Carl Zeiss, Germany).

Maintenance of stemness markers was evaluated by fixing cells in 4% PFA, blocking and permeabilizing in 3% BSA, 0.1% triton-X-100 in PBS for 15 minutes, and incubating overnight at 4°C with antibodies targeting SSEA4 and Oct-4. Cells were washed 3× 5 minutes in PBS then incubated for 1 hour in secondary antibodies. Cells were washed 3× 5 minutes in PBS, with the inclusion of 1:10000 Hoechst 33342 in the penultimate wash, before imaging using an Axio Observer Z1 inverted microscope (Carl Zeiss, Germany). Full details of antibodies used are included in Table 1.

### iMSC differentiation

An iPSC to iMSC differentiation protocol was developed by modifying that described by Sheyn et al. (38) as follows. iPSCs to be differentiated were grown to ~80% confluence on matrigel-coated 6-well plates. Cells were washed twice with DPBS and dissociated by incubating in Accutase at 37°C for 5 minutes. DMEM/F12 was added to inhibit Accutase activity and cells pelleted by spinning at 300G for 5 minutes. Cells were resuspended in induction media (IMDM (Gibco, #12440053), 20% Knockout SR (Gibco, #10828028), 0.1mM non-essential amino acids (Gibco, #11140050), and 0.1mM 2-mercaptoethanol (Gibco, #21985023)) containing 10μM RI and 1.5e4 cells per well seeded in ultra-low attachment, round bottom, 96-well plates (Corning, #7007). Cells were pelleted in the wells by spinning the plate for 3 minutes at 100G, then incubated at 37°C for 3 days to allow embryoid bodies (EBs) to form. EBs were transferred to induction media without RI in non-adherent 6-well plates (5–6 EBs per well) using wide-bore P1000 pipette tips and incubated a further 3 days. EBs were then transferred to 1% gelatin-coated 6cm dishes, gently triturating each EB with a P10 pipette tip to enhance their capacity to adhere to the plates. Initial transfer was 10–12 EBs in 1mL induction media per dish, with an extra 1mL added the following day. EBs were left for a further 2 days to adhere to the plate before media was gently replaced with iPSC-iMSC media (induction media supplemented with 10ng/mL recombinant TGFß1) and cells incubated for 3 days, during which time nascent MSCs begin to migrate out of the adhered EBs. Media was replaced with MSC media (alpha-MEM (Gibco, #12561), 1X GlutaMAX (Gibco, #35050–061), 5% FBS) and was changed twice weekly. When cells were approaching confluence, typically at the second MSC media change at D18, they were dissociated with Accutase and passaged to new 1% gelatin-coated dishes in MSC media. Again, media was changed twice weekly and when cells were confluent, Accutase was used to passage them to uncoated T25 flasks, at which point they were considered P0 iMSCs. Cells were cultured and expanded in T75 flasks, freezing cryostocks at each passage in MSC media with 10% DMSO. For routine passaging cells were dissociated with Accutase. For cryopreservation cells were frozen in MSC media supplemented with 10% DMSO.

### iMSC identity confirmation

To validate the MSC identity of generated iMSC.MetRS^L274G^ cells, surface markers were immunolabelled with fluorescently conjugated antibodies and quantified by flow cytometry. In brief, iMSC.MetRS^L274G^ cells in the passage number range of P4-P10 were dissociated with Accutase and plated 2e5 cells per well in non-tissue culture treated 96-well round-bottom plates for staining. Two panels of fluorescently conjugated antibodies were designed, one to target positive markers (CD73-APC, CD90-BV421, and CD105-PE) and one to target negative markers (CD34-APC, CD45-APC, CD11b-APC, CD79a-PE, and HLA-DR-BV421). Each panel also included SYTOX Green (Invitrogen, #S34860) as a viability marker. Full antibody details are available in Table 1. Experimental design included unstained cell groups, full stain groups for each panel, single stain controls, and fluorescence minus one (FMO) groups for each panel as required. Cells were incubated in antibodies for 30 minutes, then washed twice in 2% FBS in PBS. Approximately 15 minutes before initiation of flow cytometry, SYTOX Green (to a final dilution of 5μM) was added to the relevant samples. Samples were run on an Attune NxT flow cytometer with gates set according to single stain and FMO controls. Full antibody details are included in Table 1.

To functionally confirm MSC identity, iMSC.MetRS^L274G^ cells underwent trilineage differentiation to chondrocyte, osteoblast, and adipocyte lineages using the Human Mesenchymal Stem Cell Functional Identification Kit (R&D Systems, #SC006) according to manufacturer’s protocol. In short, iMSC.MetRS^L274G^ cells were plated in 24-well plates or 15mL Falcon tubes at the kit manufacturer’s recommended densities in the provided base medias: 2.1e4 cells/cm^2^ for adipogenic differentiation, 4.2e3 cells/cm2 for osteogenic differentiation, and 2.5e4 cells/15mL falcon tube for chondrogenic differentiation. Recommended differentiation media was replaced every 3 days and differentiated cells fixed with 4% PFA after 14 days of differentiation. Manufacturer’s protocol was followed to label for mFABP4 (adipocytes) and hOsteocalcin (osteoblasts). Chondrocyte pellets were transferred to a round-bottom 96-well plate and stained with 1% Alcian Blue 8GX solution (MP Biomedicals, #152624) overnight at room temperature. Staining solution was aspirated, chondrocytes washed 3 times in 0.1 M HCl and once in dH_2_O, and imaged on a Keyence BZ-X800E microscope.

### iMSC.MetRS^L274G^ functional validation

Taqman gene expression probes were used to confirm MetRS^L274G^ transgene expression was not silenced during differentiation from iPSC to iMSC. Since endogenous MetRS in these cells is human, and the MetRS^L274G^ transgene is murine, we were able to take advantage of species-specific probes to differentiate between endogenous and transfected MetRS expression. RNA was extracted from unmodified-, and MetRS^L274G^-expressing iPSCs and their derivative iMSCs following differentiation using the TRIzol extraction method. In brief, cells were washed in DPBS, scraped, and pelleted by centrifugation. Pellets were lysed with TRIzol reagent (Ambion, #15596018), then mixed with chloroform, followed by centrifugation to separate phases. The aqueous phase was collected and RNA precipitated with ice cold isopropanol. RNA pellet was washed with 75% ethanol, dried, and dissolved in nuclease-free-dH_2_O. Potential DNA contamination was eliminated using Qiagen RNeasy Minikit (#74106) according to manufacturer’s protocol with Qiagen RNase-free DNase (#79254). Nanodrop was used to determine RNA concentration and establish purity. cDNA was generated using High Capacity cDNA Reverse Transcription Kit (AppliedBiosystems, #4368814) and RT-PCR run using TaqMan Universal PCR Master Mix (ThermoFisher, #4304437) with FAM-tagged murine- (Mm01165889_m1 Mars) and human-specific (Hs01112701_g1 MARS) TaqMan gene expression probes (ThermoFisher Scientific). Following thermocycling, reaction products were run on 1% agarose gels containing ethidium bromide and imaged with a Chemidoc MP (BioRad) to demonstrate expression of the murine transcript exclusively in cells engineered with MetRS^L274G^ for both iPSCs and their derivative iMSCs.

To confirm the expressed *Mars* transcripts resulted in functional protein, FUNCAT was performed as described earlier.

### Assessment of prolonged inflammatory signalling in human monocytes

To model an inflammatory environment, we used THP-1 human monocytes, a kind gift from the Anastasiadis Lab, Mayo Clinic, cultured in RPMI-1640 (Gibco, #11875–093) with 10% FBS and 0.05mM 2-mercaptoethanol (Gibco, #21985–023). THP-1 cells were subcultured by passaging 1:20 in fresh media upon reaching ~8e5 cells/mL. For cryopreservation THP-1 cells were frozen in complete growth medium with 5% DMSO.

Cells were plated at 7.4e4 cells/well in 96-well plates and treated with 10μg/mL LPS or vehicle (media only). After one hour, a set of samples were collected and secreted TNF-α detected using Promega Lumit TNF-α (human) Immunoassay (#W6050) according to the manufacturer’s protocol. The remaining cells were spun down, resuspended in DPBS, spun down again, and resuspended in media without LPS. 19 hours after LPS withdrawal TNF-α secretion was assessed in these cells. A total of three biological replicates per condition (vehicle and LPS) were collected.

### Comparison of iMSC secretome when co-cultured with naïve vs LPS-treated THP-1 cells

1.5e6 cells of iMSC.MetRS^L274G^ or iMSCs from unmodified iPSCs were each plated in 15cm dishes and allowed to acclimatize for 24 hours. THP-1 cells (1e6/mL) were treated for 1 hour with 10μg/mL LPS vehicle, then washed with DPBS to remove LPS and resuspended in RPMI-1640 with 0.05mM ß-mercaptoethanol, and 4mM ANL. Media in the iMSC dishes was replaced with the LPS- or vehicle-treated THP-1 cell suspensions. After 20 hours media was collected, centrifuged, and passed through a 0.2μM filter to remove cells and debris. Protease inhibitor and RIPA buffer were added to 1X and concentrated using an Amicon filter with 10kDa cutoff.

Concentrated CM was alkylated with 50μM IA as described previously and incubated overnight with streptavidin sepharose beads to sequester endogenously biotinylated proteins. Beads were removed and unbound proteins biotinylated with 10μM DBCO-S-S-Peg3-Biotin as for previously described experiments. Excess DBCO-S-S-Peg3-Biotin was removed by cleaning samples with 7kDa cutoff Zeba spin desalting columns (ThermoFisher, #89892) and samples were incubated overnight on an L507 glass slide antibody array (Raybiotech, #AAH-BLG-1–4). The following day slides were washed, incubated with streptavidin-Cy3, and washed again according to the kit protocol. Labeled slides were imaged using an InnoScan 710AL microarray scanner (Innopsys) and relative expression levels of samples extracted by Raybiotech. To identify differentially secreted proteins, we averaged background-subtracted median intensity from duplicate antibody spots and normalized to positive control spots to correct for array performance variations. A second normalization factor calculated based on BCA-determined protein concentration of the loaded samples was applied. To mitigate background signal from non-specific binding (39), we excluded proteins from all groups if their signal from non-MetRS^L274G^-expressing groups appeared in the top tertile of expression amongst all groups. Fold change was calculated by dividing signal from MetRS^L274G^-iMSCs co-cultured with LPS-treated THP-1 cells by signal from the equivalent group co-cultured with naïve THP-1 cells. The manufacturer states that a change can be considered Significant if fold change is <0.65 or >1.5. We plotted all Significant downregulations, and the top 10 largest upregulations based on absolute fold change.

## III. Results

### Homology directed repair results in targeted incorporation of MetRS^L274G^ in H4 human neuroglioma cells.

**Figure 1:**
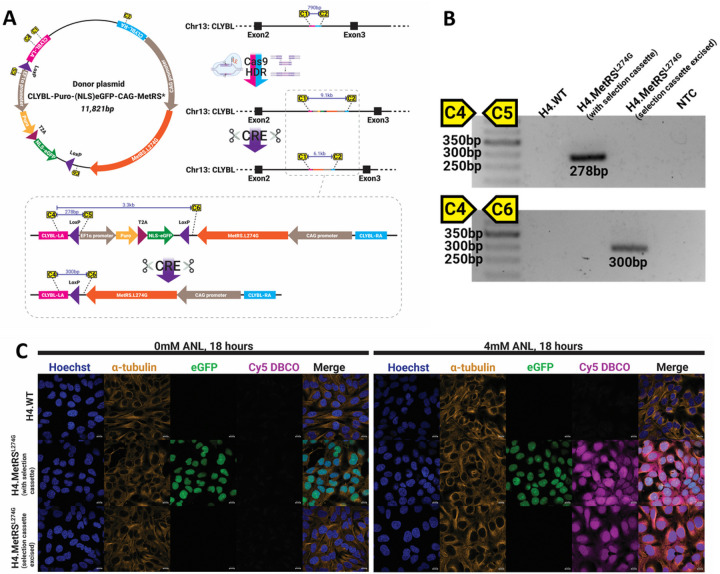
A CRISPR HDR-based strategy was designed to integrate MetRS^L274G^ into the *CLYBL* safe harbor locus of human H4 neuroglioma cells, select for single clones, and excise the selection cassette to leave minimally modified cells stably expressing MetRS^L274G^ (A). On-target integration and successful excision of selection cassette was confirmed using PCR primer-pairs designed to encompass a flanking and internal region of the donor plasmid (B). Expression of functional MetRS^L274G^ was validated by FUNCAT – H4.WT or MetRS^L274G^-expressing cells were cultured with or without 4mM ANL. Only those expressing MetRS^L274G^ and exposed to ANL-containing media displayed abundant labeling with Cy5-DBCO (C). Scale bars are 10μm.

To generate H4 neuroglioma cells stably expressing MetRS^L274G^, cells were transfected with *CLYBL*-targeting guide-RNA, recombinant Cas9, and the MetRS^L274G^ donor plasmid containing a selection cassette (Figure 1A). Clonal populations were generated from single cells and treated with recombinant TAT-CRE to excise the selection casette before again being reduced to clonal populations from single cells. PCR confirmed integration of the MetRS^L274G^ at the expected genomic locus (Figure 1B, uncropped gel in Supplementary Figure 1). Fluorescent Non-Canonical Amino acid Tagging (FUNCAT) labeling was performed to confirm functional MetRS^L274G^ expression by supplementing media with ANL, before fixing cells and incubating with Cy5 conjugated to dibenzocyclooctyne (DBCO) (Cy5-DBCO). Via the process of strain promoted cycloaddition(19), DBCO binds azide groups present on the proteins that have incorporated ANL. As expected, Cy5 fluorescence is only observed where cells both express MetRS^L247G^ and media contains ANL (Figure 1C).

### Bio-orthogonal Amino Acid Tagging (BONCAT) combined with affinity purification selectively isolates proteins from MetRS^L274G^-expressing cells co-cultured with cells expressing endogenous MetRS.

**Figure 2:**
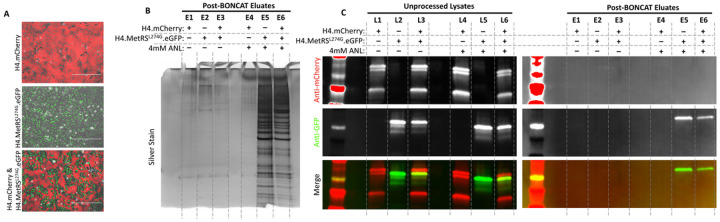
A) H4 cells expressing mCherry, or MetRS^L274G^ and eGFP were cultured separately or on the same surface. Scale bars are 400μm. Following ANL or vehicle treatment cells were lysed and subjected to BONCAT and affinity purification pipelines. B) Silver stain reveals retention of proteins exclusively from MetRS^L274G^-expressing cells that received ANL supplementation. C) WB showed specificity of retention of GFP from MetRS^L274G^-expressing cells receiving ANL, and elimination of RFP from WT cells.

To demonstrate specificity of labeling and the ability to specifically isolate proteins from MetRS^L274G^-expressing cells from mixed cultures we cultured H4 cells engineered to express mCherry (H4.mCherry), and H4 cells engineered to express MetRS^L274G^ and eGFP (H4.MetRS^L274G^.eGFP), either separately or together (Figure 2A). Following media supplementation with ANL or vehicle, cells were harvested and processed for BONCAT to biotinylate ANL-containing proteins. Biotinylated proteins were captured on streptavidin beads, stringently washed, and eluted. Eluted products were separated by electrophoresis and imaged with a silver stain. As predicted, only background labeling was observed in eluates from cells cultured in media without ANL (Figure 2B, Lanes E1, E2, and E3). From the ANL-treated cultures, H4.mCherry cell eluate also contained only background labeling (Figure 2B, Lane E4), whereas H4.MetRS^L274G^.eGFP cell eluate contained abundant labeled proteins (Figure 2B, Lane E5). For the mixed group, because wild-type and MetRS^L274G^ H4-expressing cells were co-cultured in equal numbers, approximately half of the secreted proteins came from the MetRS^L274G^ expressing cells which is demonstrated by an intermediate level of labeling seen in lane E6 of figure 2B.

To assess specificity in a more targeted manner we compared unprocessed cell lysates from the cultures described above with corresponding BONCAT-processed and affinity purified eluates by western blot (WB). In unprocessed cell lysates, mCherry expression was restricted to lanes representing lysates from cultures containing H4.mCherry cells (Figure 2C, Lanes L1, L3, L4, and L6), and eGFP was restricted to lysates from cultures containing H4.MetRS^L274G^.eGFP cells (Figure 2C, Lanes L2, L3, L5, and L6). Because BONCAT selects for proteins translated in cells expressing MetRS^L274G^ and eliminates proteins from non-MetRS^L274G^-expressing cells, eGFP was detected in post-BONCAT eluates from cultures treated with ANL, and containing H4.MetRS^L274G^.eGFP cells (Figure 2C, Lanes E5 and E6), but mCherry was undetectable in all eluates. Similarly, neither fluorescent protein was detected in eluates from cultures which did not receive ANL (Figure 2C, Lanes E1, E2, and E3). Uncropped blots can be found in Supplementary Figure 2.

### BONCAT and affinity purification can be used to isolate secreted proteins in conditioned media from MetRS^L274G^-expressing cells.

**Figure 3:**
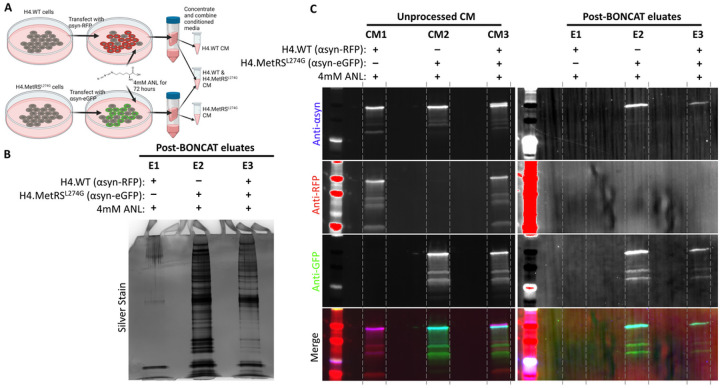
A) H4.WT cells and H4.MetRS^L274G^ cells were transiently transfected with αsyn-RFP or αsyn-eGFP, respectively. Media was supplemented with 4mM ANL for 72 hours. Conditioned media from each cell type was concentrated, and subjected to our BONCAT and affinity purification pipeline, separately, as well as after mixing in equal proportions to simulate co-culture. B) Silver stain of eluates show only minimal background labeling from H4.WT cell conditioned media, abundant protein labeling from H4.MetRS^L274G^ cell conditioned media, and intermediate labeling from the simulated co-culture conditioned media. C) Immunoblotting unprocessed concentrated conditioned media and post-BONCAT eluates for RFP and GFP shows our pipeline results in retention of GFP from the MetRS expressing cells, but not RFP from non-MetRS^L274G^-expressing cells. This demonstrates that our pipeline can be applied to secreted proteins in conditioned media.

Because MSCs are known to have a strong paracrine response, we predicted that biologically meaningful changes to the MSC proteome in response to stimuli will be most usefully detected in secreted factors from conditioned media (CM). To apply the MetRS^L274G^ system to isolation of secreted proteins from CM of co-cultures we leveraged our knowledge that overexpressed alpha-synuclein (αsyn) protein is actively secreted from cells(20). H4.WT and H4.MetRS^L274G^ cells were transiently transfected with alpha-synuclein fused to either RFP or eGFP, respectively (Figure 3A). Media was supplemented with ANL, collected after 72 hours, and concentrated. A portion of concentrated CM from each transfected cell line was combined to simulate media from co-cultured cells, and BONCAT and affinity purification was performed on all three experimental groups (H4.WT + αsyn-RFP, H4.MetRS^L274G^ + αsyn-eGFP, and H4.WT + αsyn-RFP & H4.MetRS^L274G^ + αsyn-eGFP). Silver stain of the eluates demonstrated only minimal background signal from the H4.WT + αsyn-eGFP eluate (Figure 3B, Lane E1), abundant labelled protein in the H4.MetRS^L274G^ + αsyn-RFP eluate (Figure 3B, Lane E2), and an intermediate level of protein from the combined media eluate (Figure 3B, lane E3). WB of the pre-BONCAT-concentrated CM showed immunoreactivity for αsyn in all groups (Figure 3C, Lanes CM1–3). By contrast, after BONCAT and affinity purification, no αsyn fusion proteins could be detected in the eluate from H4.WT cells (Figure 3C, Lane E1). An αSyn fusion protein was present in eluates from both the H4.MetRS^L274G^ CM, and from the 50:50 combined media, with the only fluorescent protein detected in these groups being eGFP, consistent with only proteins secreted from the MetRS^L274G^-expressing cells being retained in the eluate (Figure 3C, Lanes E2 and E3). Uncropped blots can be found in Supplementary Figure 3.

### MetRS^L274G^-based protein isolation eliminates the need for serum-free media for proteomic analyses.

A major limitation of proteomics on CM is highly abundant IgG and albumin in serum reduces detection power by masking less abundant proteins released from cells of interest. By utilizing BONCAT and affinity purification to isolate secreted proteins from cells expressing MetRS^L274G^, these contaminants are vastly reduced, allowing for the detection of proteins of interest that may otherwise be obscured without introducing additional confounding factors that result from growing cells in serum-free conditions that can affect cellular phenotype and viability. Supplementary Figure 4 shows a comparison of silver stains of ANL-supplemented CM with and without serum, demonstrating that albumin overwhelms pre-BONCAT samples (Supplementary Figure 4A) compared to samples that have undergone BONCAT and affinity purification (Supplementary Figure 4B) where the albumin signal is greatly reduced.

### Targeted insertion of functional MetRS^L274G^ into iPSCs does not alter stemness or pluripotency.

Having established the power and specificity of the mutant MetRS approach to specifically isolate proteins secreted into CM in a co-culture scenario in H4 cells, we next sought to apply it to MSCs. While some groups have demonstrated successful incorporation of MetRS^L274G^ into MSCs by viral transduction(14, 15), we used the previously described innovative CRISPR/Cas9-mediated targeted approach to generate iPSCs stably expressing MetRS^L274G^ for subsequent differentiation to iMSCs.

**Figure 4:**
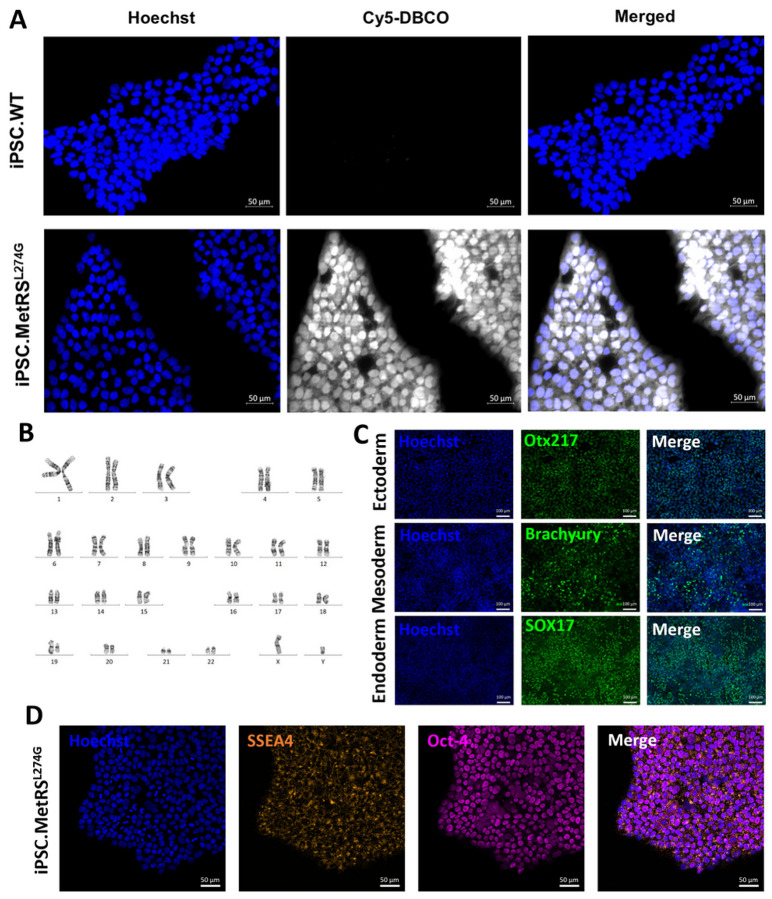
iPSC cells were stably transfected with MetRS^L274G^ and cultured in 4mM ANL-containing media. A) FUNCAT labeling with Cy5-DBCO shows labeling of translated proteins exclusively in MetRS^L274G^-expressing cells. Scale bars are 50μm. B) G-band karyotyping of edited cells shows no induction of chromosomal abnormalities. C) To demonstrate persisting pluripotency, cells were differentiated to ectoderm, mesoderm, and endoderm germ layers and labeled with Otx217, brachyury, and SOX17, respectively. Scale bars are 100μm. D) Continued stemness was validated by staining MetRS^L274G^-expressing iPSCs with SSEA4 and Oct-4. Scale bars are 50μm.

Functional MetRS^L274G^ expression in iPSCs was confirmed by FUNCAT labeling with Cy5-DBCO following ANL treatment (Figure 4A). G-band karyotyping revealed normal chromosomal architecture, validating that no abnormalities had been induced during gene editing and cell expansion (Figure 4B). Pluripotency was assessed by trilineage differentiation to 3 germ layers - ectoderm, mesoderm, and endoderm, with positive immunostaining for Otx217, brachyury, and SOX17, respectively demonstrating successful differentiation (Figure 4C). Finally, stemness was assessed and found to be preserved by immunostaining for SSEA4 and Oct-4 (Figure 4D).

### iMSCs derived from MetRS^L274G^-expressing iPSCs incorporate exogenously supplied ANL during protein translation.

**Figure 5:**
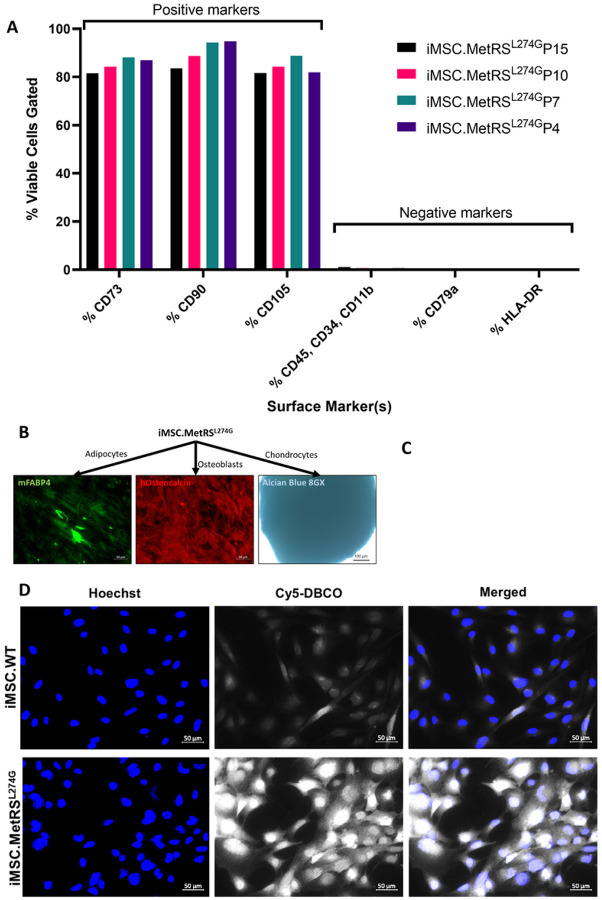
MetRS^L274G^-expressing iPSCs were differentiated to iMSCs. iMSC identity was validated by (A) expression of CD73, CD90, and CD105, and lack of expression of CD45, CD34, CD11b, CD79a, and HLA-DR, and (B) multipotency demonstrated by differentiation to adipocytes (mFABP4), osteoblasts (hOsteocalcin), and chondrocytes (Alcian Blue 8GX). C) Reverse transcription PCR with species-specific Taqman probe sets was used to confirm continued expression of MetRS^L274^ (Mars) in iMSCs following differentiation and expansion. Functionality of MetRS^L274G^ was confirmed by FUNCAT labeling with Cy5-DBCO in cells cultured in ANL-supplemented media.

Validated MetRS^L274G^-expressing iPSCs were differentiated into iMSCs using a defined media protocol. iMSC identity was confirmed by flow cytometry to detect expression of CD73, CD90, and CD105 in most cells (>80%), and absence of expression (<1% of cells) of CD45, CD34, CD11b, CD79a, and HLA-DR (Figure 5A). Trilineage differentiation to adipocytes, osteoblasts, and chondrocytes was confirmed with immunolabeling for mFABP4, hOsteocalcin, and staining for Alcian blue 8GX, respectively (Figure 5B). Previous studies have shown capacity for iPSC transgene expression to be silenced during differentiation(21). We therefore confirmed persistent MetRS^L274G^ expression in iMSCs derived from MetRS^L274G^-expressing iPSCs using reverse-transcription PCR with Taqman primers targeting a mouse specific *Mars* (*MetRS*) transcript to distinguish the mouse-derived mutant form (MetRS^L274G^), from endogenous *MARS1* (*METRS*), detected with human-specific primers (Figure 5C). FUNCAT labeling of ANL treated MetRS^L274G^-expressing iMSCs with Cy5-DBCO was used to validate that the expressed MetRS^L274G^ was indeed still functional and able to incorporate ANL into translated proteins as expected (Figure 5D).

### Profiling of the secretome of co-cultured MetRS^L274G^-expressing iMSCs to identify differentially secreted proteins in response to microenvironment changes.

As proof-of-concept in an applied manner, we co-cultured iMSCs in baseline and inflammatory environments to demonstrate that the iMSC secretome changes when subjected to different stimuli from their co-culture partners, and that we can leverage the MetRS^L274G^ expression in iMSCs to specifically detect changes elicited as a result of interactions with their environment.

**Figure 6:**
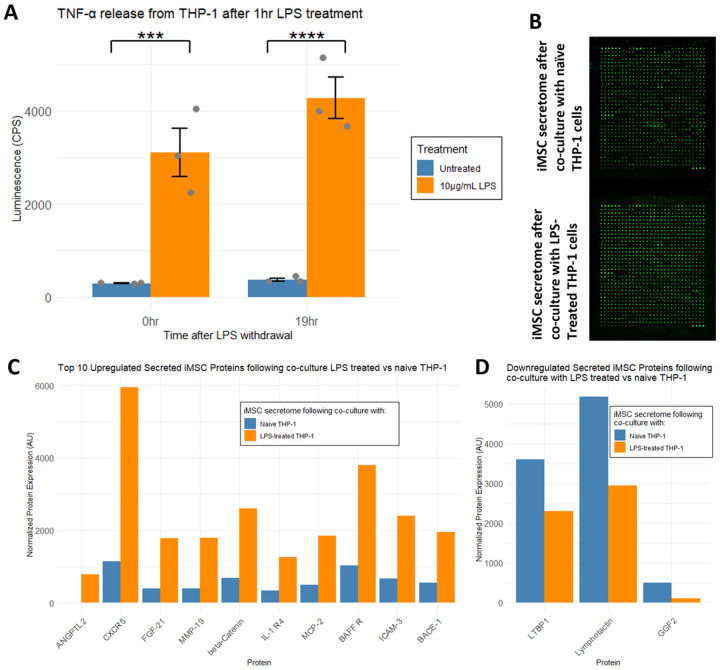
A) An inflammatory phenotype was elicited in THP-1 monocytes by treating for 1 hour with LPS. This could be measured by TNF-α release measured by Lumit assay, which persisted at least 19 hours following LPS withdrawal. B) MetRSL274G-expressing iMSCs were cultured with naïve or LPS-treated THP-1 cells. Conditioned media was evaluated using L507 antibody array to identify differently secreted iMSC-proteins. C) Top 10 upregulated proteins, and D) downregulated proteins secreted from iMSCs cultured with LPS-treated vs naïve THP-1 cells.

We first demonstrated that lipopolysaccharide (LPS)-treated THP-1 monocytes enter an inflammatory state that persists beyond withdrawal of LPS, by showing elevated TNFα secretions compared to untreated cells 19 hours after a 1hr LPS treatment (Figure 6A). Subsequently, THP-1 cells treated with vehicle or LPS for 1 hour were washed and added to plates containing MetRS^L274G^-expressing iMSCs with ANL-containing media. CM was harvested, concentrated, and alkylated. iMSC-secreted proteins were biotinylated using DBCO-S-S-Peg3-biotin and incubated with an L507 antibody array (RayBiotech). Bound proteins were then detected with streptavidin-cy3, imaged (Figure 6B), and relative abundance between groups quantified. Here we show that in response to an inflammatory environment (co-culture with LPS-treated THP-1 cells), iMSCs secrete ANGPTL2, as well as increase their secretion of multiple proteins including CXCR5, FGF-21, MMP-19, and BACE-1, relative to levels during co-culture with naïve THP-1 cells (Figure 6C). We were also able to detect a downregulation in secretion of three proteins, LTBP1, Lymphotactin, and GGF-2, compared to iMSCs cultured in baseline conditions (Figure 6D). This experiment nicely demonstrates the capacity of MetRS^L274G^-expressing iMSCs to have their secretomes profiled in experimentally manipulated environmental conditions, with powerful detection of the most relevant signals and no contamination from other microenvironment components.

## IV. Discussion

Here we describe a novel technical approach to profile the secretome of co-cultured cells without interference from confounding factors found in media components, or released from the partner cell line(s) in the culture. There are two particularly innovative aspects to the methodology described herein. First, the utilization of stably expressed MetRS^L274G^ to incorporate the non-canonical amino acid, ANL, exclusively into proteins translated in these cells, and second, the application of mutant MetRS technology to iPSCs which can then be differentiated into cell types of interest while maintaining the transgene expression. Together, this study demonstrates a novel application of NCAT to characterization of the secretome from iMSCs.

The use of NCAT to label translated proteins has historically been used to good effect in cell culture models to dissect temporal events, and/or consequences of treatments(9, 10). A relatively recent development however is the use of mutated forms of MetRS to restrict tagging to proteins translated in cells expressing that mutant form, whilst not tagging proteins in cells that only express endogenous MetRS isoforms(11, 12), thereby dissecting the contribution of a specific cell type in mixed cultures. There are limited alternative approaches to dissect a co-cultured cell’s broad proteome in this way. One possible approach to achieve similar results would be to dissociate co-cultured cells from the culture surface and use fluorescence-activated cell sorting (FACS)(22) or immunopanning (IP)(23) to separate the cell type of interest before lysing and processing for proteomics. This has notable limitations including the dependence on suitable surface marker(s) for cell isolation as well as the time-consuming nature and the potential to influence cell behavior and protein stability via FACS or IP. A second limitation is that FACS or IP does not allow for the deconvolution of secreted proteins in the CM of a mixed culture environment. An alternative that could be applied to CM would be to use xenogeneic cell types for co-culture followed by in silico approaches to separate proteins based on species-specific motifs after performing proteomics on the complete CM. This again is afflicted with major limitations, not least the fact that many peptides have high conservation between species making determination of their origin cell impossible. Furthermore, when simulating a physiological niche, xenogeneic co-cultures will rarely be the most representative model.

Utilizing MetRS^L274G^ overcomes the limitations described above with some caveats. First, only proteins containing methionine residues will be tagged. Because the first methionine is frequently cleaved post-translationally(24), internal methionines are required for reliable tagging by the MetRS^L274G^ system, therefore excluding those proteins with no internal methionines and N-terminal methionine cleavage. This may not be a major concern since 88% of the 81,791 proteins annotated as part of the human proteome in the UniProtKB database contain at least 1 internal methionine residue, rising to 96% when examining only the 20,423 reviewed proteins (UP000005640, accessed 04/04/2023)(25). Another potential confound is cell toxicity associated with long term ANL treatment. It is suggested to carefully titrate ANL concentration in media to balance adequate labeling with avoiding adverse effects on cell proliferation(15). In our experience for <72-hour treatment paradigms, 4mM ANL did not appear to have harmful effects. There are also multiple studies that apply ANL-based NCAT in vivo and in vitro without any observable increase in toxicity(26, 27). Traditional NCAT approaches culture cells in media lacking the canonical amino acid to be replaced (methionine). However, for co-cultures, the partner cell line is unable to utilize the surrogate non-canonical amino acid (ANL) and requires complete media containing methionine for protein translation to proceed. In the presence of complete media, methionine competes with ANL for incorporation into proteins translated in MetRS^L274G^-expressing cells, making it imperative that: i) ANL is provided in excess at a concentration that favors its incorporation preferentially over methionine, and ii) MetRS^L274G^ is abundantly expressed in every desired cell. The addition of excess ANL to media addresses the competition for ANL incorporation by methionine, and to ensure MetRS^L274G^ is expressed in all cells we designed a strategy to generate clonal lines of cells that stably express MetRS^L274G^ under the control of a strong, constitutively active cytomegalovirus/chicken beta actin (CAG) promoter(28). Not only did this ensure we have universal expression of MetRS^L274G^, the guided CRISPR-HDR approach minimizes concerns regarding disruption of the genome from off-target insertions, which is a distinct advantage over viral transduction approaches utilized by other groups(14, 15).

Another major advantage conferred by the approach described herein is the ability to separate MetRS^L274G^-expressing cell derived proteins from serum proteins. Highly abundant media components such as albumin and IgG can mask the less abundant, cell-secreted proteins when fetal bovine serum (FBS) or similar supplements are included in the media, but culturing cells in starved conditions is not optimal and is reported to result in a decrease in secreted proteins(29, 30). While there are albumin-depletion methodologies, such as incubation with Cibacron Blue(31), they are expensive, lack specificity, and typically result in the loss of additional protein beyond the undesired media components. Since media components have not had the opportunity to incorporate ANL, they are excluded when following the BONCAT pipeline and affinity purification protocol, eliminating their masking effect on the lower abundance, MSC-secreted proteins. This means the co-culture can be carried out in optimal conditions, without the need to starve cells of nutrients.

A second major innovation to the approach detailed herein is its application to induced-MSCs, derived from genetically-edited iPSCs. The main motivation for taking this approach was to establish the stable, targeted expression of MetRS^L274G^ in iMSCs to facilitate the characterization of the iMSC secretome in response to specific stimuli. The generation of clonal lines with targeted integration, ensures uniform expression throughout the population and avoids concerns about off-target gene insertion. Performing the gene editing in iPSCs before differentiating to iMSCs means the valuable early MSC passages(32) are preserved for co-culture, not used during the editing, validation of MetRS^L274G^ expression, or expansion of cells.

As well as facilitating the genetic modification of cells, this approach offers further potential benefits. There is a known heterogeneity in the performance of primary MSC batches, even from the same donor(33). With controlled differentiation of the cells, and the capacity to generate very large batches, we mitigate heterogeneity by generating large stocks at low passage. With the limitless passaging potential of iPSCs, we can now generate a theoretically unlimited supply of iMSCs(18). Not only this, but if interest extends beyond MSCs, protocols exist for differentiation of iPSCs to a multitude of other cell types. One important consideration for this is that the stability of the promotor must be robust enough to avoid silencing during differentiation. Our initial attempts to develop this system using the EF1α promoter to drive MetRS^L274G^ expression resulted in transgene expression being gradually silenced during iPSC passaging, and particularly during differentiation to iMSCs, consistent with other reports(21). This issue was resolved using the CAG promoter, but will need to be assessed when differentiating to alternative cell types.

Another important consideration for the use of iMSCs vs primary MSCs, is whether they are truly analogous and can be used interchangeably. There is already literature demonstrating differences between the expression signature of MSCs depending on their source tissue(34), and similarly between iMSCs and primary cells(18). Though the ISCT definition of MSCs states more than 95% of cells should express CD73, CD90, and CD105, and fewer than 1% should express CD34, CD45, CD11b, CD79a, or HLA-DR(35), these markers are a moving target within the field with no definitive profile for what is categorically an MSC. Further, groups report differing levels of positive and negative markers depending on source of cells(36). Those we generate here have an MSC-like profile, although not strictly adhering to the ISCT criteria. Rather than an over-reliance on narrow surface marker criteria to define whether a cell is an MSC, perhaps more crucial is the functional capacity. Here we show the functional capacity of iMSCs to differentiate into the three lineages expected of MSCs(35). Further by using this platform to generate iMSCs from iPSCs, which are abundantly available from banking services or can be generated relatively non-invasively from individuals, the simple differentiation protocol makes it possible to screen large numbers of iPSC-derived iMSCs to identify the most potent in responding to the co-culture disease model of choice. Furthermore, by having the ability to isolate the iMSC-derived proteome, it may be possible to identify a signature for a ‘potent’ MSC for a particular condition by comparing the proteome of effective and non-effective MSC lines.

## V. Conclusions

To conclude, here we combine precise genome editing of iPSCs to generate cells stably expressing MetRS^L274G^ and facilitate the specific profiling of proteomes from a cell-type of interest within a mixed culture environment. This is effective on both cell lysate, and on secreted proteins in CM. Further, we demonstrate the application using induced-MSCs, a potentially powerful tool in understanding the innate regenerative capacity of the human body.

## Figures and Tables

**Table 1:** Antibodies used in this manuscript.

Antibody	Source	Dilution
Hoechst 33342	Invitrogen (H3570)	1:10,000 (Immunocytochemistry (ICC))
Alpha-Tubulin (mouse)	Millipore-Sigma (T9026)	1:500 (ICC)
Green Fluorescent Protein (rabbit)	Proteintech (50430-2-AP)	1:2,000 (Western blot (WB))
Red Fluorescent Protein [6G6] (mouse)	Chromotek (6g6)	1:1,000 (WB)
α-synuclein [MFJR1] (rabbit)	Abcam (ab138501)	1:2,000 (WB)
Otx217 (goat)	R&D Systems (967331), from StemXVivo Ectoderm Kit (SC031B)	1μg/100μL (ICC)
Brachyury (goat)	R&D Systems (967332), from StemXVivo Mesoderm Kit (SC030B)	1μg/100μL (ICC)
Sox17 (goat)	R&D Systems (967330), from StemXVivo Endoderm Kit (SCO19B)	1μg/100μL (ICC)
SSEA4	Cell Signaling (#4755)	1:500 (ICC)
Oct-4	Cell Signaling (#2750)	1:200 (ICC)
CD73 Monoclonal Antibody (AD2), APC	eBioscience (17-0739-41)	1:20 (Flow cytometry (FC))
Brilliant Violet 421^™^ anti-human CD90 (Thy1) Antibody	Biolegend (328121)	1:20 (FC)
CD105 (Endoglin) Monoclonal Antibody (SN6), PE	eBioscience (12-1057-41)	1:20 (FC)
CD45 Monoclonal Antibody (HI30), APC	eBioscience (17-0459-41)	1:20 (FC)
CD34 Monoclonal Antibody (4H11), APC	eBioscience (17-0349-41)	1:20 (FC)
CD11b Monoclonal Antibody (ICRF44), APC	eBioscience (17-0118-41)	1:20 (FC)
CD79a Monoclonal Antibody (HM47), PE	eBioscience (12-0792-41)	1:20 (FC)
Brilliant Violet 421^™^ anti-human HLA-DR Antibody	Biolegend (307635)	1:20 (FC)
Sytox Green	Invitrogen (S34860)	30nM (1:1,000) (FC)
mFABP4 (goat)	R&D Systems (967799), from Human Mesenchymal Stem Cell Functional Identification Kit (SC006)	10μg/mL (ICC)
hOsteocalcin (goat)	R&D Systems (967801), from Human Mesenchymal Stem Cell Functional Identification Kit (SC006)	10μg/mL (ICC)
Goat anti-Mouse IgG (H+L) Cross-Adsorbed Secondary Antibody, Alexa Fluor^™^ 568	Invitrogen (A-11004)	1:500 (ICC)
IRDye^®^ 800CW Goat anti-Rabbit IgG Secondary Antibody	Licor (925-32211)	1:10,000 (WB)
IRDye^®^ 680RD Goat anti-Mouse IgG Secondary Antibody	Licor (925-68070)	1:10,000 (WB)
FlexAble CoraLite^®^488 Antibody Labeling Kit for Rabbit IgG	ProteinTech (KFA001)	1μL/0.5μg Ab (WB)
Goat anti-Mouse IgG (H+L) Highly Cross-Adsorbed Secondary Antibody, Alexa Fluor^™^ Plus 555	Invitrogen (A-32727)	1:500 (ICC)
Goat anti-Rabbit IgG (H+L) Highly Cross-Adsorbed Secondary Antibody, Alexa Fluor^™^ 647	Invitrogen (A-21245)	1:500 (ICC)
Donkey anti-Goat IgG (H+L) Cross-Adsorbed Secondary Antibody, Alexa Fluor^™^ 488	Invitrogen (A-11055)	1:1,000 ICC
Donkey anti-Mouse IgG (H+L) Highly Cross-Adsorbed Secondary Antibody, Alexa Fluor^™^ 568	Invitrogen (A-10037)	1:1,000 ICC

Purpose is indicated by the following abbreviations: ICC = Immunocytochemistry, WB = Western Blot, FC = Flow cytometry

## Data Availability

The datasets generated and/or analyzed during the current study are available from the corresponding author on reasonable request.

## Abbreviations

αsyn: alpha-synuclein
ANL: azidonorleucine
BCA: bicinchoninic acid
BONCAT: bioorthogonal non-canonical amino acid tagging
BP: base pairs
BSA: bovine serum albumin
CAG: cytomegalovirus/chicken beta actin promoter
CLYBL: citramalyl-CoA lyase
CM: conditioned media
DBCO: dibenzocyclooctyne
DTT: dithiothreitol
EB: embryoid body
eGFP: enhanced GFP
FACS: fluorescence-activated cell sorting
FBS: fetal bovine serum
FC: flow cytometry
FMO: fluorescence minus one
FUNCAT: fluorescent non-canonical amino acid tagging
HDR: homology directed repair
IA: iodoacetamide
ICC: immunocytochemistry
IMDM: Iscove Modified Dulbecco Media
iMSC: induced mesenchymal stromal cell
IP: immunopanning
iPSC: induced pluripotent stem cell
LF: low fluorescence
LPS: lipopolysaccharide
MetRS: methionyl-tRNA synthetase
MetRS^L274G^: *mutant* methionyl-tRNA synthetase
MCS: multiple cloning site
MSC: mesenchymal stromal cell
MWCO: molecular weight cutoff
NCAT: non-canonical amino acid tagging
NLS: nuclear localization signal
PCR: polymerase chain reaction
PVDF: polyvinylidene fluoride
RFP: red fluorescent protein
RI: rock inhibitor Y-27632
RNA: ribonucleic acid
TBS: tris-buffered saline
WB: western blot
